# Angiokeratoma of Fordyce—A rare complication of laser hair removal: A case report of two patients

**DOI:** 10.1002/ccr3.9077

**Published:** 2024-06-14

**Authors:** Fatemeh Moeineddin, Elnaz Pourgholi, Mohammad Rahmati‐Roudsari, Reza M. Robati

**Affiliations:** ^1^ Skin Research Center Shahid Beheshti University of Medical Sciences Tehran Iran

**Keywords:** adverse effects, alexandrite laser, angiokeratoma, case report, laser hair removal

## Abstract

**Key Clinical Message:**

This study highlights the first documented cases of angiokeratoma of Fordyce following laser hair removal (LHR) emphasizing the importance of patient selection and careful laser use. It underscores the importance of understanding LHR‐associated risks, particularly for patients with darker skin. The efficacy of topical rapamycin as an alternative treatment for angiokeratomas is also discussed.

**Abstract:**

Laser hair removal (LHR) has emerged as a widely accepted method for achieving long‐term hair reduction. While generally considered safe, it is important to study the possible adverse events to optimize patient care. Here, we present a unique case report of angiokeratoma of Fordyce, a rare vascular lesion, following LHR. Two patients experienced the development of these lesions subsequent to LHR treatment sessions, characterized by a severe burning sensation during the procedure. Interestingly, both individuals exhibited varicose veins on their legs, suggesting a potential risk factor for this complication. Our findings highlight the importance of understanding the mechanisms underlying LHR‐induced adverse events and the need for further research to elucidate associated risk factors and management strategies. This case report serves to enhance awareness among clinicians and emphasizes the significance of patient counseling regarding the potential side effects of LHR.

## 1 INTRODUCTION

In many cultures, unwanted hair remains a common aesthetic concern. Hypertrichosis and hirsutism can both have an impact on psychological well‐being by contributing to anxiety and depression.^1^ The predominant method for hair removal, laser treatment, has emerged as the gold standard due to its longer‐lasting results compared to alternative approaches.^1^ One of the most widely used laser systems is the Alexandrite 755 nm laser. Lighter skin types are better suited for this laser system because it absorbs less melanin pigment and penetrates the skin deeper.^2^ Conversely, the Nd: YAG Laser, emitting pulses at 1064 nm wavelength and exhibiting minimal melanin absorption, is considered safer for individuals with darker skin tones.^3^ In modern practice, these laser types are integrated into systems to maximize effectiveness and reduce side effects.^4^

Since laser hair removal (LHR) is not a standardized process and the parameters of LHR are individualized, it is important to have precise laser selection, optimal pulse duration, and appropriate fluence to achieve maximum efficacy and safety.^5^ Consequently, background information on the principles of selective photothermolysis and the practical application of laser‐tissue interactions is essential for safe LHR practice.^6^

While LHR is generally considered safe, common adverse events such as transient erythema, perifollicular edema, and temporary pain may occur. These adverse events depend on some variables, including the operator's knowledge, skin type, treatment site, laser system, and parameter set.^1^ Unfortunately, LHR can be practiced by untrained or unsupervised non‐physician providers, raising the possibility of preventable complications.^7^ In this report, we present two cases of angiokeratoma of Fordyce following LHR, marking the first instances documented in the literature.

## 2 CASE PRESENTATION

### 2.1 Case 1

**FIGURE 1:**
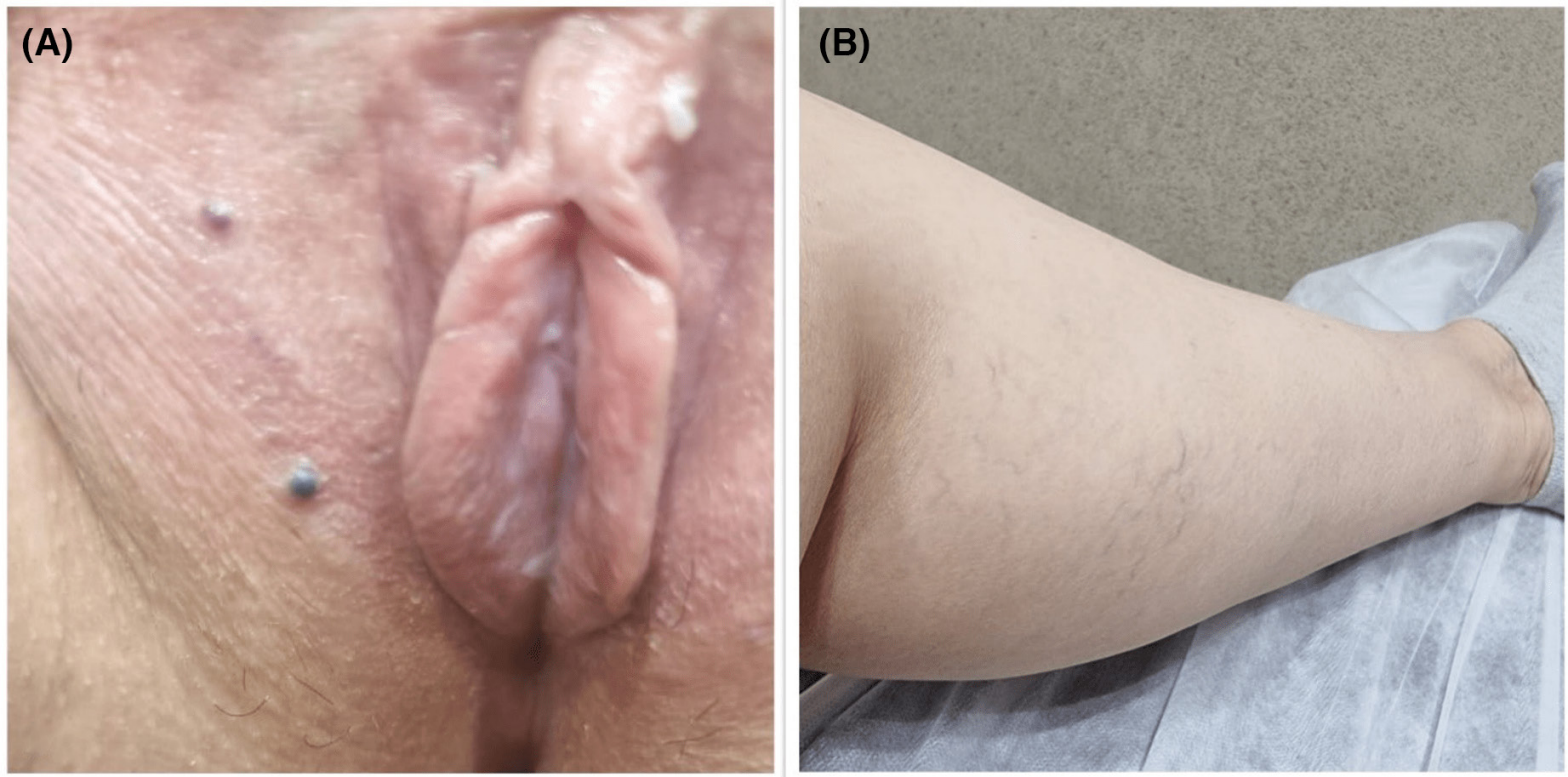
Two well‐defined, dusky erythematous papules on the labia major (A). Varicose veins on the medial side of the shin (B).

A 29‐year‐old woman with Fitzpatrick type III skin presented at our clinic complaining of two raised purple‐blue lesions on her labia major which had been present for a month. She first noticed these asymptomatic papules 2 months post an LHR procedure using the Alexandrite 755 nm laser (The Elite+™ Aesthetic Workstation, Cynosure, Westford, MA). The patient received LHR for her genital area every 6 weeks for a total of five sessions with the setting of 18–20 J/cm^2^ fluence, a 20–30 ms pulse duration, and a spot size of 18 mm. After four sessions, due to an incomplete response, the operator adjusted the pulse duration to 20 ms, resulting in an intense burning sensation during treatment, without subsequent burn or blister formation. Despite being diagnosed and treated for genital warts, no improvement was noted. The patient had no notable medical, surgical, or habitual history. Upon physical examination, two well‐defined, dusky erythematous papules were evident on her labia major, with no signs of scaling or erosion (Figure 1). Associated lymphadenopathy or mucosal lesions were not observed.

### 2.2 Case 2

**FIGURE 2:**
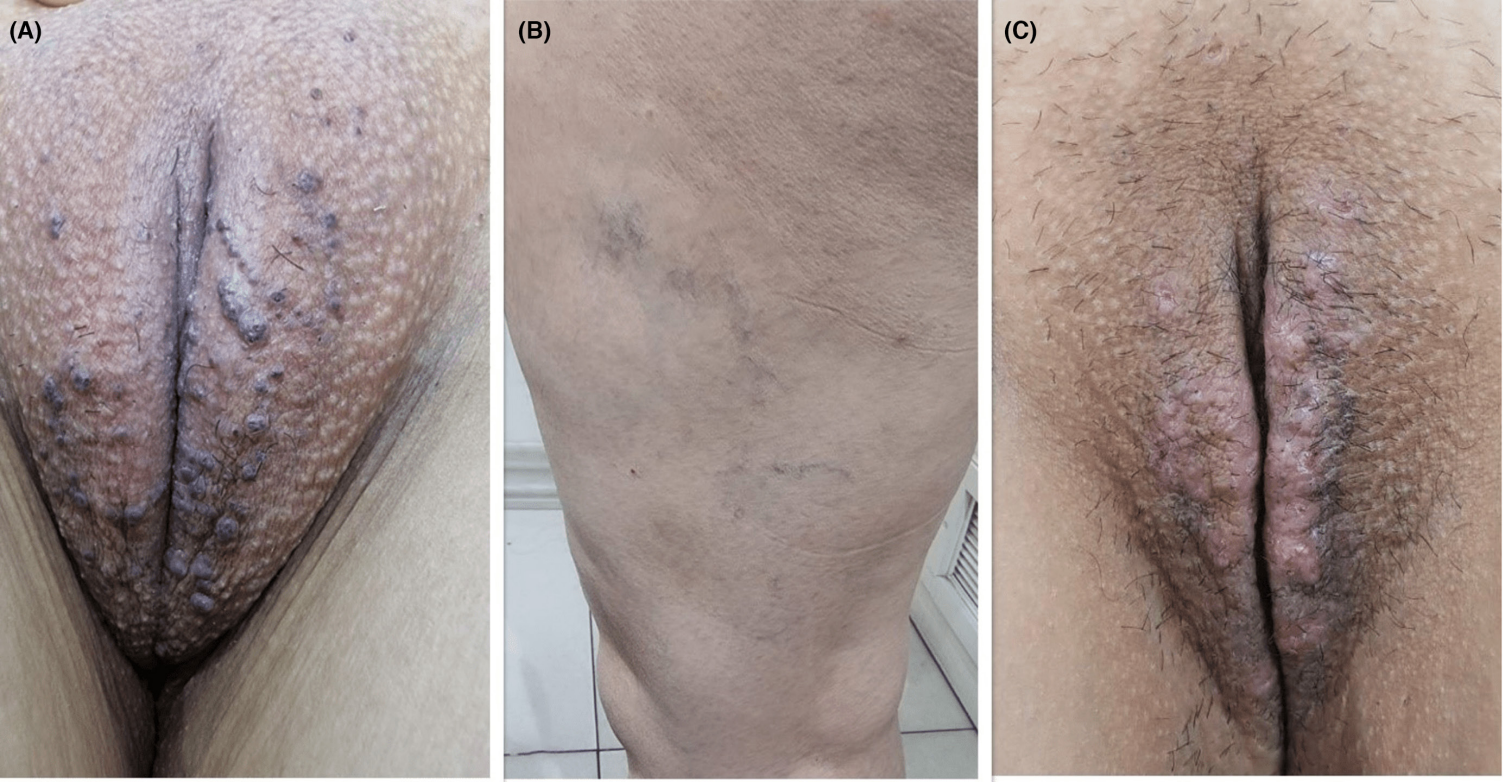
Multiple purple‐blue papules on the labia major (A). Varicose veins on the thigh (B). One month after treatment with rapamycin 0.25% cream twice daily (C).

A 32‐year‐old female of Fitzpatrick type III skin presented with several asymptomatic purplish papules on her labia major. She had previously undergone LHR treatment for her genital area using a long pulse Alexandrite 755 nm laser (The Elite+™ Aesthetic Workstation, Cynosure, Westford, MA). The treatment consisted of six sessions every 6 weeks, delivering 18 J/cm^2^ with an 18 mm spot size and a pulse duration of 20–30 ms. However, during the final session, she noted the inadequate response of unwanted hair, prompting an increase in fluence to 20 J/cm^2^ while keeping other parameters unchanged. She experienced a severe burning sensation during this session, although no burning or blistering occurred afterward. About 2 months post the final LHR session, she noticed multiple bumps on the treated areas, leading to initial treatment for clinically suspected genital warts, which showed no improvement. Her medical, surgical, drug, and habitual history was unremarkable. Physical examination revealed multiple purple‐blue papules on the labia major with a fine scale (Figure 2). Varicose veins were noted on the medial aspect of her thighs. Other mucocutaneous and systemic examinations were normal.

## 3 METHODS

### 3.1 Case 1

Based on the patient's medical history and physical examination, a punch biopsy was obtained from one of the lesions. Differential diagnoses included pyogenic granuloma, angiokeratoma, and cherry hemangioma. Subsequent punch biopsy revealed multiple dilated, thin‐walled, congested capillaries in the papillary dermis, along with varying degrees of hyperkeratosis, rete ridge elongation, and acanthosis in the epidermis, consistent with angiokeratoma of Fordyce. Systemic evaluation unveiled varicose veins on both legs, with no other underlying conditions such as HPV infection, sexually transmitted infections (STI), vulvar varicosity, or hemorrhoids detected. As the lesions remained asymptomatic, the patient declined further treatment.

### 3.2 Case 2

**FIGURE 3:**
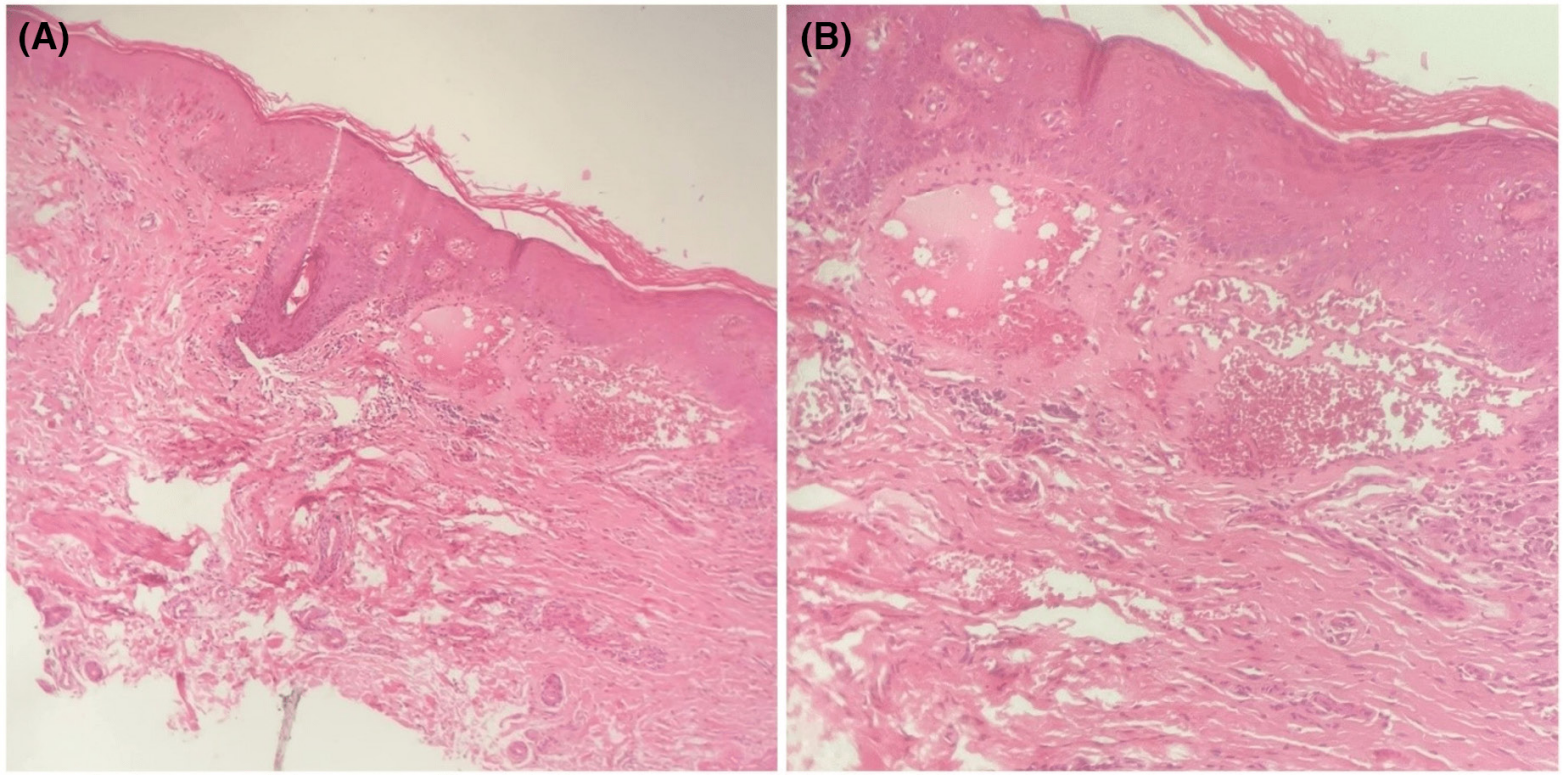
Histopathologic slides from a punch biopsy of Case 2. Note the presence of epidermal acanthosis and multiple dilated thin‐walled vessels filled with red blood cells throughout the papillary dermis, which is consistent with angiokeratoma of Fordyce. (A, B; Hematoxylin–eosin stain; original magnifications: A × 20, B × 40).

A punch biopsy was performed showing epidermal acanthosis and hyperkeratosis, along with multiple dilated thin‐walled vessels filled with red blood cells throughout the papillary dermis, consistent with angiokeratoma of Fordyce (Figure 3). Diagnostic studies, including screening for STI, pap smear, and abdominopelvic ultrasound, were performed to rule out any underlying disease all of which returned negative results including for HPV infection. Subsequently, she was treated with topical rapamycin 0.25% cream twice daily for 3 months, leading to a near‐complete resolution of the condition.

## 4 CONCLUSION AND RESULTS

Both patients were diagnosed with angiokeratoma of Fordyce after undergoing LHR, resulting in the cessation of LHR treatment sessions. Case 1 refused further treatment and the patient was lost to follow‐up. Conversely, Case 2 received treatment with rapamycin 0.25% cream applied twice daily and was monitored monthly. Despite experiencing minimal erythema and irritation at the lesion sites, the treatment was well tolerated. Remarkably, significant improvement was noted after 3 months. The patient remains under ongoing treatment and observation.

## 5 DISCUSSION

The popularity of LHR has surged in recent years; however, it is important to be aware of the associated risks.^8^ The majority of side effects are mild and transient; typically manifesting as erythema, perifollicular edema, and intraoperative pain.^9^ Nonetheless, there is a potential for more severe side effects such as blisters, hyperpigmentation, persistent hypopigmentation, and permanent scarring.^10^ Individuals with darker skin phototypes and recent sun exposure are particularly susceptible to adverse reactions post‐photo epilation.^9^ Therefore, careful patient selection is crucial, especially considering that darker skin phototypes (IV–VI) and tanned individuals are at higher risk of experiencing vesiculation, dyspigmentation, and/or scarring.^11^

Purpura, a rare side effect of LHR, has been documented in approximately 7% of patients.^12^ Since LHR systems target the melanin in hair follicles, there exists a risk of epidermal or dermal injury during treatment, potentially impacting vascular structures.^13^ This article presents the first reported cases of angiokeratoma of Fordyce, another vascular side effect following LHR.

Angiokeratoma is a benign vascular proliferation with hyperkeratosis on the surface.^14^ It is believed to stem from degenerative changes in elastic tissue within blood vessels, leading to elevated local venous pressure. Predisposing factors include increased parity, obesity, hemorrhoids, pelvic inflammatory disease, pregnancy, and previous hysterectomy.^14^, ^15^ Furthermore, long‐term human papillomavirus infections have been associated with vulvar angiokeratomas.^16^ Considering that angiokeratoma of Fordyce can occur as a result of tissue inflammation, it is reasonable to expect the manifestation of these lesions following LHR.

The long‐pulse alexandrite laser has been utilized in two case reports to treat angiokeratoma circumscriptum.^17^, ^18^ This effectiveness is speculated to be due to the strong absorption of laser energy by deoxyhemoglobin at the lesion site. However, our current research also suggests a paradoxical effect, where the laser treatment may trigger the development of angiokeratomas. This paradoxical action highlights the complexity of laser‐tissue interactions and the need for further research to understand the mechanisms behind these effects. As previously described as “paradoxical hypertrichosis” this dual nature of the alexandrite laser—both therapeutic and potentially triggering—should be carefully considered in clinical practice to optimize patient outcomes and minimize adverse effects.^19^

In the current report, both patients experienced a severe burning sensation during their final Alexandrite 755 nm laser treatment session, following adjustments made to enhance epilation efficacy. However, neither patient reported blistering or burning post‐procedure. Interestingly, varicose veins were observed on the legs of both individuals, leading to speculation that it may be a risk factor for this side effect. With no history of other diseases and diagnostic studies revealing no underlying abnormalities, we conclude that the emergence of vascular lesions in the genital area was likely a consequence of LHR. Excessive thermal damage and resultant epidermal and dermal injury, similar to the reported process in LHR‐induced purpura, could explain the development of these lesions.

Treating angiokeratomas can be particularly challenging, especially when dealing with more extensive lesions in sensitive areas like the labia major. Various treatment options have been documented, such as surgical excision, electrodessication, cautery, cryotherapy, and laser therapy.^20^ Recently, rapamycin has emerged as an effective treatment for vascular tumors and malformations by inhibiting mTOR, and reducing VEGF production and cellular proliferation.^21^ Topical rapamycin is generally well‐tolerated, with mild to moderate irritation as the most common side effect. Concerns about immunosuppression are minimal due to its high molecular weight, limiting skin absorption.^22^

To our knowledge, this is the second documented case of angiokeratoma of Fordyce successfully treated with topical rapamycin.^23^ This highlights its potential as a promising alternative treatment for patients with angiokeratoma of Fordyce who cannot tolerate other therapeutic options.

This study underscores the necessity for laser operators to possess a comprehensive understanding of how lasers interact with the skin tissue and the potential risks associated with photoepilation prior to administering laser‐assisted hair removal. Furthermore, it emphasizes the importance of counseling patients to foster realistic expectations regarding the treatment process.

## 6 CONCLUSION

LHR stands as a prevalent method for eliminating unwanted hair. Understanding potential side effects is pivotal to preventing unnecessary investigations and enhancing management strategies. To the best of our knowledge, this is the first report of angiokeratoma of Fordyce after LHR. These vascular lesions develop as a result of thermal injury to the skin's vascular structures. Further research is required to identify the environmental factors, patient characteristics, and risk factors associated with these adverse events.

## AUTHOR CONTRIBUTIONS

**Fatemeh Moeineddin:** Conceptualization; methodology; supervision. **Elnaz Pourgholi:** Data curation; investigation; writing – original draft. **Mohammad Rahmati‐Roudsari:** Data curation; supervision. **Reza M. Robati:** Writing – review and editing.

## FUNDING INFORMATION

This research received no specific grant from funding agencies in the public, commercial, or not‐for‐profit sectors.

## CONFLICT OF INTEREST STATEMENT

The authors declare no conflicts of interest.

## ETHICS STATEMENT

The study was performed according to the Declaration of Helsinki guidelines and the patients provided written informed consent to publish any associated data and accompanying images.

## CONSENT

Written informed consent was obtained from the patient to publish this report in accordance with the journal's patient consent policy.

## Data Availability

All study data can be obtained upon request from the authors.
